# Calcium-dependent regulation of antioxidant metabolism in maize seedlings under cadmium stress

**DOI:** 10.1515/biol-2025-1313

**Published:** 2026-06-01

**Authors:** Xian Shi, Xun Li, Tiantao Wang, Qiong Kong, Yanhua Tang, Hengling Meng

**Affiliations:** School of Agronomy, Honghe College, 661199, Mengzi, China

**Keywords:** antioxidant system, cadmium stress, calcium, maize seedlings

## Abstract

Calcium (Ca^2+^) is an essential nutrient for plants, contributing to their improved tolerance against heavy metal stress. This study aimed to investigate how varying Ca^2+^ concentrations (0, 0.5, 2, and 5 mM) regulate maize seedling growth, physiological and biochemical traits, and cellular antioxidant defense systems under cadmium (Cd) stress, using hydroponic experiments to elucidate the mechanisms underlying Cd toxicity alleviation. The results revealed that Ca^2+^ supplementation mitigated the inhibitory effects of Cd stress on root length, dry weight, and photosynthetic pigment contents. Also, Ca^2+^ application increased the activities of superoxide dismutase and catalase, as well as the contents of glutathione and ascorbic acid in leaves, with maximum increases of 14.9 %, 65.39 %, 146 %, and 135 %, respectively, compared with the control (CK) treatment. Peroxidase activity significantly decreased under Ca^2+^ treatment, with a maximum reduction of 34.38 % compared with that under CK treatment, whereas malondialdehyde content markedly increased, reaching 19.84 % higher than that under CK treatment. Meanwhile, principal component analysis revealed that the 5 mM Ca^2+^ treatment achieved the highest composite score, indicating optimal antioxidant capacity in maize seedlings and reduced membrane lipid peroxidation caused by excessive Cd accumulation. This study enhances the understanding of the role of Ca in plant stress responses and provides insights for safe maize production in Cd-contaminated environments. However, given Cd accumulation in plants, whether the maize used in this study is suitable for animal and human consumption requires further investigation.

## 1 Introduction

The farmland soil environment is a crucial factor influencing crop yield and quality. The rapid industrialization in China has led to the emergence of heavy metal pollution as a prominent environmental concern. Cadmium (Cd) is characterized by high mobility and a high exceedance rate in soil monitoring sites. Hence, it readily migrates from farmland to crops, subsequently entering the food chain and posing significant risks to human health. It is therefore recognized as one of the most hazardous heavy metals [1]. Cd absorbed through roots or foliage accumulates in plant tissues, interfering with diverse physiological and biochemical processes. This accumulation leads to metabolic disorders, growth inhibition, and severe oxidative stress [2]. The detrimental effects of Cd on plants include suppression of seed germination, structural damage to roots and cytoskeletal microtubules, inhibition of photosynthesis, and disruption of cellular redox systems [3]. Cd stress reduces wheat growth, yield, and photosynthetic efficiency while increasing malondialdehyde (MDA) and hydrogen peroxide (H_2_O_2_) contents [4]. Furthermore, excessive reactive oxygen species (ROS), including H_2_O_2_, OH^−^, O_2_^−^, and O_2_, are generated under high Cd concentrations, inducing membrane lipid peroxidation and ultimately impairing crop growth [5].

Plants alleviate Cd toxicity by enhancing the activities of antioxidant enzymes, including superoxide dismutase (SOD), peroxidase (POD), catalase (CAT), ascorbate peroxidase (APX), and glutathione reductase (GR), as well as nonenzymatic antioxidants such as glutathione (GSH), ascorbic acid (AsA), mannitol, and flavonoids. These mechanisms prevent excessive ROS accumulation and maintain cellular redox balance [6]. A study on *Aster subulatus* revealed that despite a significant reduction in biomass under Cd stress, the pronounced increase in POD and CAT activities allowed the plant to sustain normal growth and demonstrate effective detoxification capabilities [7]. Calcium (Ca^2+^) enhances POD and SOD activities, promoting the elimination of intracellular peroxides via antioxidant groups. This process reduces MDA content by suppressing lipid peroxidation, enhances plasma membrane stability, and alleviates Cd-induced cytotoxicity in rice cells [8].

Ca^2+^ is an essential nutrient for plant growth. It plays a crucial role in regulating physiological and biochemical processes and maintaining cellular functions. As a second messenger, Ca^2+^ enhances plant resilience to abiotic stress through stress signaling pathways, thereby exerting protective and growth-promoting effects [9], 10]. Exogenous Ca^2+^ application mitigates Cd toxicity by alleviating Cd-induced physiological impairments, such as root growth inhibition, ROS accumulation, oxidative damage, and root tip cell death [11]. Exogenous Ca^2+^ supplementation promotes plant height and biomass accumulation under varying Cd stress levels. Concurrently, it increases MDA content while reducing antioxidant enzyme activities and Cd content in various plant tissues [12]. Furthermore, the accumulation and distribution of Cd within plants are influenced by the concentration and chemical forms of exogenous Ca^2+^. Therefore, investigating the specific mechanisms through which exogenous Ca^2+^ regulates growth and Cd detoxification in maize seedlings is imperative.

Maize, a staple crop extensively cultivated in China, serves as both a primary forage resource and a vital industrial feedstock. Given its high tolerance to diverse environmental stressors, it has been widely used as a key crop species in studies on heavy metal contamination [2], 13]. Recent studies have demonstrated that Ca^2+^ supplementation alleviates Cd-induced photosynthetic decline in maize and effectively reduces oxidative damage in seedlings under both Cd stress and low-temperature stress [14]. In addition to alkaline earth metals such as Ca, exogenous small molecular compounds have also been verified to alleviate Cd stress in maize seedlings. For instance, exogenous glycerol application was found to regulate the antioxidant defense system, photosynthetic metabolism and ion homeostasis in maize seedlings, thereby significantly mitigating Cd-induced growth inhibition and oxidative damage [15]. This finding enriches the technical system of chemical regulation for Cd stress alleviation in maize, and provides a comparative basis for evaluating the regulatory effects of different exogenous substances on maize under Cd stress.

The escalating Cd contamination in agricultural soils has caused substantial crop yield losses. Based on the dual roles of exogenous Ca^2+^ in regulating plant growth and alleviating Cd-induced toxicity, and considering its crucial multifunctional roles in plant growth and metabolic processes, we hypothesized that Ca^2+^ supplementation could effectively mitigate Cd-induced adverse effects on maize seedling growth. This study investigated the effects of varying Ca^2+^ concentrations on maize seedling growth, physiological and biochemical parameters, and antioxidant enzyme activities under Cd stress, aiming to determine the extent to which exogenous Ca^2+^ alleviates Cd toxicity. Given the current lack of mechanistic understanding of Cd toxicity mitigation, this study provides valuable insights for exploring potential molecular mechanisms underlying heavy metal stress alleviation in diverse plant species in the future.

## 2 Materials and methods

### 2.1 Seedling treatment

The maize variety used in this study was the Chl “Hongdan 3,” developed through multi-generation selection at the Honghe Prefecture Agricultural Research Institute, Yunnan, China. Seeds with plump kernels and uniform size were selected. The seeds were washed with deionized water, soaked in tap water, germinated in the dark, and sprouted in vermiculite. The maize seedlings that grew uniformly were transplanted to a solar greenhouse and cultivated with a 1/2 Hoagland nutrient solution (pH 6.5). The composition was half of each component listed in Table 1.

**Table 1:** Composition of improved Hoagland nutrient solution.

Chemical nomenclature	Mass concentration (mg·L^−1^)	Chemical nomenclature	Mass concentration (mg·L^−1^)	Chemical nomenclature	Mass concentration (mg·L^−1^)
KNO_3_	506	NH_4_NO_3_	80	MgSO_4_	493
KI	0.83	KH_2_PO_4_	136	H_3_BO_3_	6.2
FeSO_4_·7H_2_O	13.9	MnSO_4_	22.3	C_10_H_14_N_2_Na_2_O_8_·2H_2_O	18.65
ZnSO_4_	8.6	CuSO_4_	0.0025	CoCl_2_	0.0025
Na_2_MoO_4_·2H_2_O	0.25	Ca(NO_3_)_2_·4(H_2_O)	945	–	–

### 2.2 Selection of Cd concentrations

Different concentrations of Cd chloride were used (0, 50, 100, 150, 200, 250, 300, and 350 μM). The preliminary results showed that 250 μM Cd inhibited the growth and germination of 50 % of maize seedlings.

### 2.3 Experimental design

After growing in 1/2 Hoagland nutrient solution for 5 days, the plants were transferred to containers filled with full-strength Hoagland solution (composition shown in Table 1). They were subjected to the following treatments: Control (CK): 0 μM Cd; K-1: 0.5 mM Ca + 250 μM Cd; K-2: 2 mM Ca + 250 μM Cd; and K-3: 5 mM Ca + 250 μM Cd. Both Cd and Ca were added simultaneously to the solution. A total of 40 L of nutrient solution was placed in lidless rectangular containers, with three plants cultivated per 200 mL of solution, resulting in 60 plants in total. The experiment included three replicates. The hydroponic experiment was conducted in an artificial climate chamber with a temperature of 25 °C, a light period of 08:00–18:00, and a humidity of 60 %. Samples were collected after 30 days of treatment.

### 2.4 Plant sample collection and measurements

The plant materials in the seedling stage (four-to six-leaf stage; 30 days post-emergence) were collected to assess growth parameters, physiological and biochemical traits, and antioxidant defense system enzymes. The seedlings were rinsed with distilled water, and the growth attributes were recorded. Maize leaf samples were frozen at −80 °C for subsequent analysis of physiological/biochemical traits and antioxidant activity. The sampled seedlings were oven-dried at 70 °C until constant weight to determine root dry weight (RDW) and shoot dry weight.

### 2.5 Growth parameter measurements

Stem length was measured from the base to the tip of the top leaf for each plant. The roots were carefully removed from the nutrient solution to record root length (RL).

### 2.6 Determination of the contents of photosynthetic pigments

The chlorophyll content was determined using the method proposed by Arnon [16], whereas the carotenoid content was measured using the method proposed by Davis [17]. Leaf samples (0.1 g) were homogenized in 5 mL of 80 % (*v*/*v*) acetone and filtered. The absorbance was recorded at 645, 663, and 480 nm using an ultraviolet spectrophotometer (UV2700; Shimadzu, Japan). The contents of photosynthetic pigments were calculated as follows:

$$\text{Chl }a\;\left(\text{mg}/g\text{ fresh weight}\right)=\left[12.7\left({\text{OD}}_{663}\right)\;–\;2.69\left({\text{OD}}_{645}\right)\right]\times V/\left(1000\;\times W\right)$$

$$\text{Chl }b\;\left(\text{mg}/g\text{ fresh weight}\right)=\left[22.9\left({\text{OD}}_{645}\right)\;–\;4.68\left({\text{OD}}_{663}\right)\right]\times V/\left(1000\;\times W\right)$$

$$\text{Carotenoids }\left(\text{mg}/g\text{ fresh weight}\right)=[\left({\text{OD}}_{480}+0.114\left({\text{OD}}_{663}\right)\;–\;0.638\left({\text{OD}}_{645}\right)\right]/2500$$

where *V* represents the volume of acetone (mL), and *W* denotes the fresh weight (g) of the leaf sample.

### 2.7 Determination of MDA content

MDA content was determined via the thiobarbituric acid method [18]. Briefly, 0.1 g of tissue was homogenized in 10 % trichloroacetic acid (TCA) and centrifuged at 12,000 *g* for 10 min. Subsequently, 2 mL of supernatant was mixed with 2 mL of 10 % TCA in a fresh centrifuge tube, boiled for 15 min, and cooled on ice. After centrifugation at 3000 *g* for 10 min, the absorbance of the supernatant was measured at 532 and 600 nm.

### 2.8 Determination of antioxidant enzyme activities

Enzyme extraction: The leaf samples (0.1 g) were ground in a pre-cooled mortar with ice-cold 50 mM phosphate buffer (pH 7.0, containing 1 % Polyvinylpyrrolidone, 100 μM EDTA, and 5 mM Dithiothreitol). The homogenate was centrifuged at 12,000 *g* for 20 min at 4 °C, and the supernatant was collected as the crude enzyme extract.

SOD activity was assayed by the nitroblue tetrazolium (NBT) photoreduction method [19], with one unit defined as the amount of enzyme required to inhibit 50 % of NBT photoreduction.

POD activity was measured via the guaiacol colorimetric method [20], where one unit corresponds to a Δ*A*_470_ of 0.01 per minute. CAT activity was determined by monitoring the decrease in absorbance at 240 nm, with one unit defined as a reduction of 0.1 absorbance units per minute.

### 2.9 Determination of oxidant content

GSH content was measured by the DTNB method [20]. Samples (0.5 g) were divided into two portions: one for dry weight determination and the other for GSH extraction. The tissue was homogenized in 5 mL of 5 % trichloroacetic acid (TCA) and centrifuged at 15,000 *g* for 10 min. The supernatant was adjusted to 5 mL. A reaction mixture containing 0.25 mL of supernatant, 2.6 mL of 150 mM NaH_2_PO_4_ (pH 7.7), and 0.18 mL DTNB reagent was incubated at 30 °C for 5 min, and the absorbance was measured at 412 nm.

Ascorbic acid content was determined following Nino and Shah [21]. Fresh maize tissue (0.1 g) was ground with thiobarbituric acid (TBA) and centrifuged at 10,000 *g* for 10 min. An aliquot (500 μL) of the supernatant was mixed with 2 mL of diluted sulfuric acid, incubated at 37 °C for 30 min, and centrifuged at 12,000 *g*. The absorbance was measured at 520 nm using a spectrophotometer (Hitachi U-2910; Tokyo, Japan).

### 2.10 Determination of Cd and Ca^2+^ contents in plants

Dried shoots and maize roots were ground to a fine powder. Samples (0.2 g) were digested with 2 mL of H_2_O_2_ and 6 mL of HNO_3_ in a microwave digestion system (Labtech, Ethos One, Milestone Srl) under the following conditions: preheating for 10 min, heating at 190 °C for 15 min, and cooling for 20 min. The digestate was diluted to 50 mL with deionized water. Cd and Ca^2+^ contents were quantified using an inductively coupled plasma optical emission spectrometer (ICP-OES; PerkinElmer, USA). The Cd translocation factor was calculated as the ratio of Cd accumulation in shoots to that in roots [22].

### 2.11 Data processing

Prior to one-way analysis of variance (ANOVA), normality was tested using the Shapiro–Wilk test, and homogeneity of variance was verified using Levene’s test. The data were statistically analyzed and visualized using Excel 2017. One-way ANOVA with Duncan’s test (*P* < 0.05) was performed in IBM SPSS Statistics 26. Correlation analysis, principal component analysis (PCA), and data visualization were performed using the R language.

## 3 Results and analysis

### 3.1 Effects of different Ca^2+^ concentrations on the growth characteristics of maize seedlings under Cd stress

The plant height, RL, above-ground dry weight (ADW), and RDW of maize seedlings increased with increasing Ca^2+^ concentrations under Cd stress, reaching the highest values under the K-3 treatment (1.99 %, 10.60 %, 15.72 %, and 3.64 % higher than that under CK treatment, respectively) (Table 2). The RL and ADW under K-3 treatment differed significantly (*P* < 0.05) from those under CK, K-1, and K-2 treatments, whereas the plant height and RDW showed no significant difference compared with those under CK treatment. The plant height, RL, and ADW were significantly lower under K-1 treatment than under CK treatment, although RDW reduction was not statistically significant. These results indicated that low Ca^2+^ concentrations exacerbated Cd stress, whereas high Ca^2+^ concentrations mitigated Cd toxicity.

**Table 2:** Effects of different Ca^2+^ concentrations on the growth and photosynthetic pigments in maize under Cd stress.

Treatment	Shoot length (cm)	Root length (cm)	Shoot dry weight (g)	Root dry weight (g)
CK	45.27 ± 0.23a	31.98 ± 0.23c	15.46 ± 0.26b	3.57 ± 0.13a
K-1	43.55 ± 0.15b	30.75 ± 0.36b	14.23 ± 0.32c	3.39 ± 0.19a
K-2	45.19 ± 0.81a	33.72 ± 0.09b	16.03 ± 0.38b	3.47 ± 0.09a
K-3	46.17 ± 0.20a	35.37 ± 0.13a	17.89 ± 0.31a	3.70 ± 0.11a

Data represent the mean ± SD (*n* = 3). In columns, different letters indicate significant differences among treatments (*P* < 0.05).

### 3.2 Effects of different Ca^2+^ concentrations on photosynthetic pigment content in maize seedlings under Cd stress

The chlorophyll a, chlorophyll b, and carotenoid contents in Ca^2+^-treated seedlings exhibited an initial decrease followed by an increase with increasing Ca^2+^ concentrations under Cd stress (Table 3). The chlorophyll a content under all Ca treatments was significantly lower (*P* < 0.05) than that under CK treatment, with K-2 treatment showing the lowest value (15.94 % reduction). The chlorophyll b content under all treatments, except the K-2 treatment, was significantly higher (*P* < 0.05) than that under the CK treatment, peaking under the K-1 treatment (42.05 % increase). The carotenoid content under K-1 and K-2 treatments was significantly lower than that under CK treatment, with K-2 treatment showing the lowest value (22.27 % reduction). Overall, 5 mM Ca^2+^ treatment (K-3) enhanced chlorophyll a and carotenoid accumulation.

**Table 3:** Effects of different Ca^2+^ concentrations on photosynthetic pigments in maize seedlings under Cd stress.

Treatment	Chl a (mg g^−1^ FW)	Chl b (mg g^−1^ FW)	Caro. (mg g^−1^ FW)
CK	14.37 ± 0.10a	3.02 ± 0.12b	2.20 ± 0.10a
K-1	13.17 ± 0.30b	4.13 ± 0.06a	1.83 ± 0.11b
K-2	12.08 ± 0.37c	2.96 ± 0.18b	1.71 ± 0.11b
K-3	13.11 ± 0.68d	4.29 ± 0.13a	2.25 ± 0.12a

Data represent the mean ± SD (*n* = 3). In columns, different letters indicate significant differences among treatments (*P* < 0.05). Chl a, chlorophyll a; Chl b, chlorophyll b; Caro, carotenoids.

### 3.3 Effects of different Ca^2+^ concentrations on MDA content in maize under Cd stress

**Figure 1:**
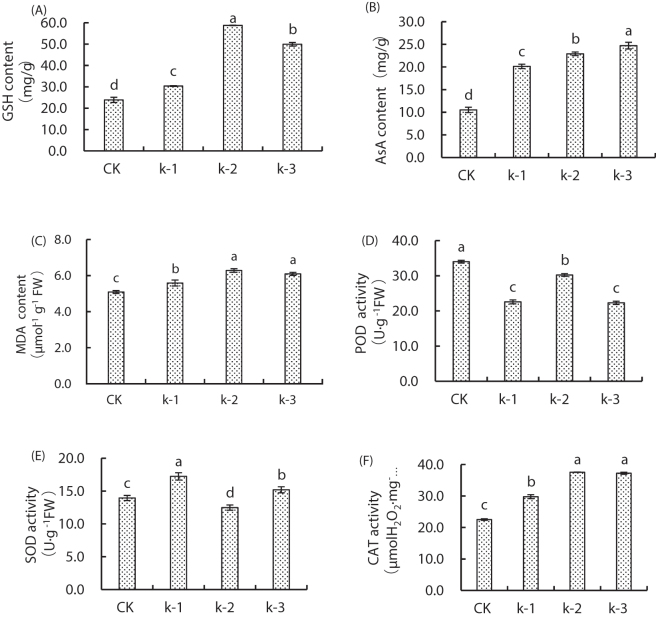
Effect of calcium (Ca^2+^; CK: 0 mM Ca^2+^ + 0 μM Cd; K-1: 0.5 mM Ca^2+^ + 250 μM Cd; K-2: 2 mM Ca^2+^ + 250 μM Cd; K-3: 5 mM Ca^2+^ + 250 μM Cd) and cadmium (Cd) treatments on (A) glutathione (GSH), (B) ascorbic acid (AsA), and (C) malondialdehyde (MDA) contents and (D) peroxidase (POD), (E) superoxide dismutase (SOD), and (F) catalase (CAT) activities in maize seedlings. Data represent the mean ± SD (*n* = 3). Different letters indicate significant differences among treatments (ANOVA, Duncan’s test, *P* < 0.05).

Significant differences (*P* < 0.05) in GSH, AsA, and MDA contents were observed among treatments (Figure 1). The GSH content under all Ca^2+^ treatments was significantly higher than that under CK treatment, peaking under K-2 treatment (146 % increase, Figure 1A). The AsA content increased significantly (*P* < 0.05) under all Ca^2+^ treatments compared with CK treatment, with K-3 treatment showing the highest value (135 % increase, Figure 1B). The MDA content was significantly elevated (*P* < 0.05) under Ca^2+^ treatments compared with CK treatment, reaching a maximum under K-2 treatment (23.44 % increase), although no significant difference existed in the contents between K-2 and K-3 treatments (Figure 1C).

### 3.4 Antioxidant enzyme activities

POD activity in Ca-treated seedlings under Cd stress was significantly lower (*P* < 0.05) than that under CK treatment, showing an initial increase followed by a decline with rising Ca^2+^ concentrations. K-2 treatment exhibited the highest POD activity (11.13 % reduction vs CK treatment), whereas K-3 treatment showed the lowest activity (34.38 % reduction). No significant difference was observed between K-1 and K-2 treatments (Figure 1D).

SOD activity decreased initially and then increased with increasing Ca^2+^ concentrations, peaking under K-1 treatment (23.68 % increase vs CK treatment), whereas K-2 was significantly lower than CK (Figure 1E).

CAT activity was significantly higher (*P* < 0.05) under all Ca treatments compared with CK treatment, peaking under K-2 treatment (66.80 % increase), with no significant difference between K-2 and K-3 treatments (Figure 1F).

### 3.5 Cd and Ca accumulation in plant tissues under varying Ca^2+^ concentrations

**Figure 2:**
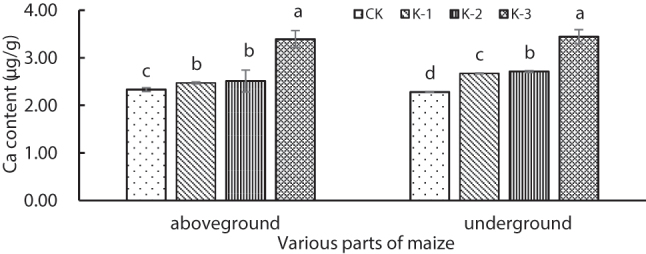
Ca^2+^ concentration in the above-ground parts and roots of maize seedlings under Cd stress at different Ca^2+^ concentrations. Data represent the mean ± SD (*n* = 3). Different letters indicate significant differences among treatments (ANOVA, Duncan’s test, *P* < 0.05).

Cd concentration in shoots and roots increased significantly (*P* < 0.05) with Ca^2+^ supply, peaking under K-3 treatment (3.43 and 20.71 μg g^−1^, respectively). The Cd translocation factor followed the order: K-2 > K-3 > K-1, with the value under K-1 treatment being 29.14 % lower than that under K-2 treatment, indicating that low Ca^2+^ concentration inhibited Cd translocation from roots to shoots (Table 4). The 5 mM Ca^2+^ treatment significantly increased (*P* < 0.05) Ca^2+^ concentration in shoots and roots by 48.05 % and 52.42 %, respectively, compared with that under CK treatment (Figure 2).

**Table 4:** Effects of Cd content and Cd transport rate in different parts of maize at different Ca^2+^ concentrations under Cd stress.

Treatment	Cd content (μg g^−1^)		Cd transport coefficient (IF)
	Above-ground parts	Roots	Above-ground parts/Cd roots
CK	0.00 ± 0.00d	0.00 ± 0.00d	0.0000 ± 0.0000d
K-1	2.18 ± 0.04c	18.13 ± 0.31b	0.1201 ± 0.0025b
K-2	3.37 ± 0.11b	19.88 ± 0.21c	0.1695 ± 0.0005a
K-3	3.43 ± 0.10a	20.71 ± 0.14a	0.1656 ± 0.0010a

Data represent the mean ± SD (*n* = 3). In columns, different letters indicate significant differences among treatments (*P* < 0.05).

### 3.6 Correlation matrix

**Figure 3:**
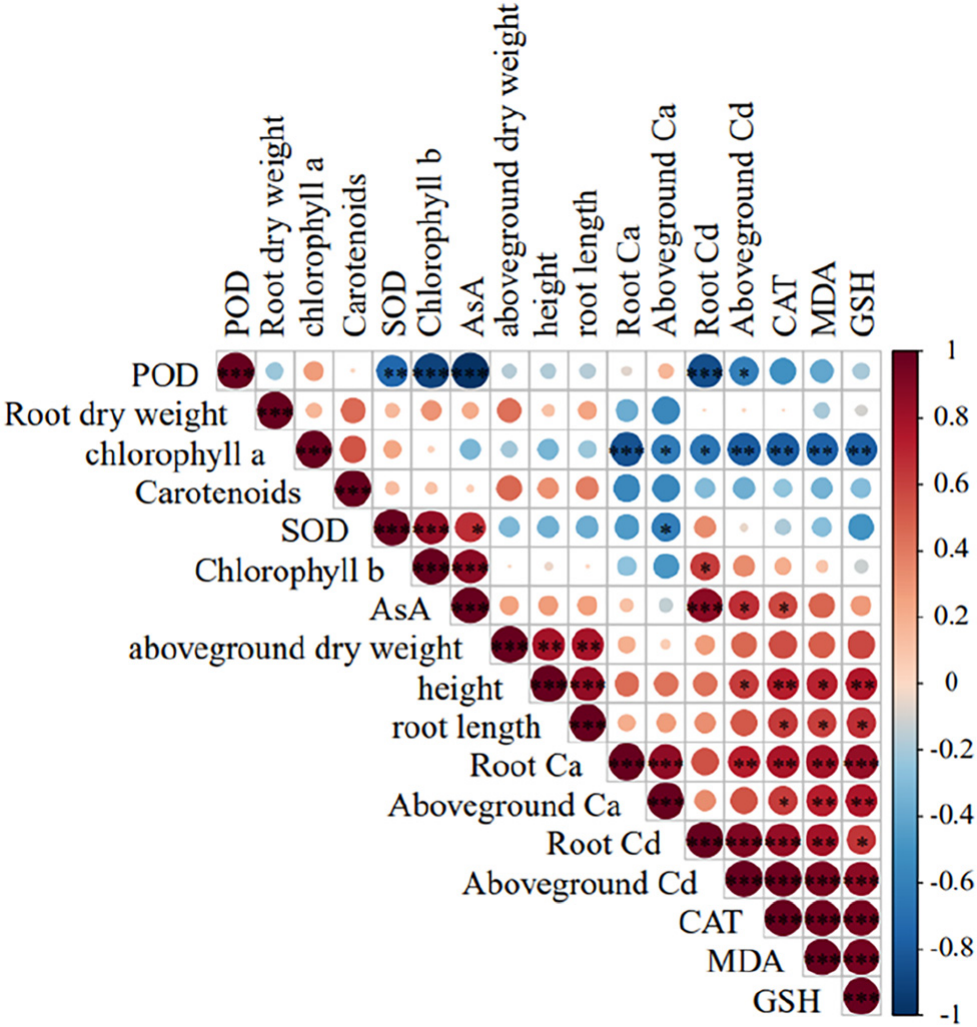
Correlation between morphological and physiological–biochemical traits of maize seedlings under Cd stress.

POD activity exhibited a highly significant positive correlation (*P* < 0.001) with chlorophyll b, AsA, and root Cd contents under Cd stress. Chlorophyll a content showed a significant positive correlation (*P* < 0.001) with root Ca^2+^ content, shoot Cd content, and CAT activity, as well as MDA and GSH contents. SOD activity was significantly positively correlated (*P* < 0.001) with chlorophyll b content. AsA content displayed a highly significant positive correlation (*P* < 0.001) with root Cd content. CAT activity showed a significant negative correlation (*P* < 0.001) with MDA and GSH contents. Root Ca^2+^ content was negatively correlated (*P* < 0.001) with shoot Ca^2+^ and GSH contents and negatively correlated (*P* < 0.01) with shoot Cd content, CAT activity, and MDA content. Shoot Ca^2+^ content exhibited a significant negative correlation (*P* < 0.01) with MDA and GSH contents. Root Cd content was negatively correlated (*P* < 0.001) with shoot Cd content, CAT activity, and MDA content. Shoot Cd content showed a highly significant negative correlation (*P* < 0.001) with CAT activity and MDA and GSH contents (Figure 3).

### 3.7 Principal component analysis

**Figure 4:**
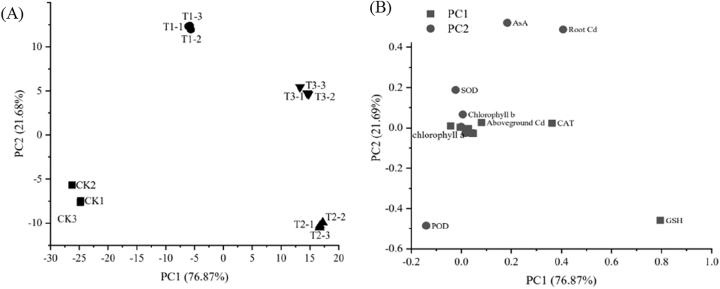
PCA of growth and physiological and biochemical indexes of maize at different Ca^2+^ concentrations under Cd stress. (A) Score plot of PCA; (B) loading plot of PCA. PC1: First principal component; PC2: second principal component.

PCA results revealed substantial differences in the effects of Ca^2+^ treatments on maize seedling growth and physiological traits. The first principal component (PC1) accounted for 76.87 % of the variance, whereas the second principal component (PC2) explained 21.69 % of the variance, cumulatively contributing 98.56 % of the total variance under different Ca^2+^ treatments (Figure 4A). The physiological and biochemical traits of maize seedlings varied markedly across treatments. For PC1, GSH content exhibited the highest contribution rate, followed by CAT activity. For PC2, root Cd content contributed most significantly, followed by AsA content (Figure 4B). The comprehensive evaluation showed that the 5 mM Ca^2+^ treatment ranked the highest, indicating its pronounced impact on photosynthetic pigment content and antioxidant metabolism in maize under Cd stress.

## 4 Discussion

Cd adversely impacts plant growth and development. It readily accumulates in plant tissues, causing physiological and metabolic disorders, growth inhibition, and severe oxidative stress [2]. As an essential mineral element for plants, Ca^2+^ also functions as a versatile signaling ion, playing crucial roles at multiple sites within various signaling cascade networks. Consequently, Ca^2+^ enhances plant stress resistance [22], 23]. The exogenous application of Ca^2+^ can improve plant adaptability to abiotic stresses and mitigate damage caused by adverse conditions [24]. which has been verified in the physiological and biochemical regulation of maize seedlings under Cd stress in this study from the perspectives of growth characteristics, photosynthetic metabolism and antioxidant defense system.

The redox balance in plants is disrupted under heavy metal stress, leading to excessive production of ROS, which triggers membrane lipid peroxidation and damages the cellular membrane system [25]. To counteract oxidative damage, plants employ both enzymatic (SOD, POD, Polyphenol Oxidase, Ascorbate Peroxidase, and Glutathione Reductase) and nonenzymatic (AsA–GSH cycle) mechanisms to scavenge excess ROS. Enzymes such as SOD, POD, and CAT exhibit strong ROS-scavenging capacity under normal conditions, enabling the timely removal of ROS. The production of free radicals in plant cells increases significantly under stress. These free radicals initiate membrane lipid peroxidation, leading to membrane damage and reduced activity of ROS-scavenging enzymes. GSH and AsA are essential small-molecule antioxidants in plants. Together, they constitute the nonenzymatic antioxidant system and participate in cellular redox processes [26]. AsA is catalyzed by APX to convert H_2_O_2_ into harmless H_2_O_2_, whereas GSH is regenerated via GR to eliminate ROS. Additionally, GSH promotes the synthesis of nonprotein thiols and phytochelatins (PCs), thereby inactivating free metal ions [27]. Exogenous Ca^2+^ application enhances SOD and CAT activities in tomatoes, increases AsA and GSH contents in *Stachys lanata*, and alleviates Cd stress, aligning with the findings of this study. POD catalyzes the decomposition of peroxides (including H_2_O_2_) and participates in lignin biosynthesis [28]. Cd transport is influenced by xylem stability [29]. POD activity may indirectly impact Cd accumulation. This study revealed a highly significant positive correlation between POD activity and root Cd content, as well as a significant positive correlation with shoot Cd content, as well as a significant positive correlation with shoot Cd content. Medium-to-high Ca^2+^ treatments enhanced SOD and CAT activities, increased the contents of antioxidants (e.g., GSH and AsA), and improved membrane stability. This might be attributed to the Ca^2+^–calmodulin complex regulating enzyme activities (SOD, POD, and CAT) or Ca^2+^ reducing Cd’s ability to displace metal ions at enzyme active sites, thereby mitigating Cd-induced inhibition of antioxidant systems.

MDA is a by-product of lipid peroxidation in metabolic processes and biological membrane systems. Its content reflects the degree of lipid peroxidation in the cell membrane [30]. An interesting phenomenon was observed in this study: Ca^2+^ treatment increased MDA content in maize seedlings under Cd stress, this is inconsistent with the traditional stress-alleviating physiological responses such as the reduction of MDA content in both shoots and roots of maize by foliar application of glycerol [15]. Thus, we proposed several potential mechanisms to explain this result based on the measured physiological and biochemical parameters. First, the experimental Ca^2+^ concentration may be a key factor. Previous studies have shown that appropriate Ca^2+^ concentrations can alleviate oxidative damage, but excessive Ca^2+^ can disrupt cellular homeostasis and induce secondary stress [31]. In this study, the Ca^2+^ concentration used (up to 5 mM) might have exceeded the optimal range for maize seedlings under Cd stress, leading to increased MDA production. Second, the treatment time (30 days) might have contributed to this phenomenon. Long-term Cd stress can cause persistent damage to the antioxidant system. Although Ca^2+^ enhances antioxidant enzyme activities and the contents of nonenzymatic antioxidants, it may not fully compensate for the cumulative oxidative damage, resulting in continued MDA accumulation [32]. Additionally, species-related differences cannot be ignored. Different plant species or varieties have varying responses to Ca^2+^ and Cd interactions. Maize variety “Hongdan 3” may have unique physiological characteristics leading to increased MDA content under Ca treatment [33].

In this study, 5 mM Ca^2+^ treatment significantly increased chlorophyll b content in maize seedlings; the carotenoid content showed no significant change (Table 3), and chlorophyll a exhibited a downward trend. These results were consistent with the findings of Alrashidi et al. [32], where foliar application of Ca^2+^ fertilizer on tomatoes significantly enhanced chlorophyll b content. This phenomenon might be attributed to exogenous Ca^2+^ regulating the activity of chlorophyll b synthase or acting as a signaling molecule to promote chlorophyll b synthesis [33]. In this study, chlorophyll a content showed a significant positive correlation with CAT activity and GSH content, whereas chlorophyll b content was positively correlated with AsA content, indicating a mutual influence of the photosynthetic and antioxidant systems. This was because photosynthetic pigments not only participate in photosynthesis but also eliminate ROS in plant cells, thereby alleviating oxidative stress [34]. Furthermore, the results of this study showed that the supplemental application of Ca^2+^ fertilizer in agricultural practices, particularly in Cd-contaminated farmlands, improved the photosynthetic performance of maize seedlings and enhanced their productivity under heavy metal stress conditions.

Relevant studies have shown that the addition of exogenous Ca^2+^ can reduce Cd content and its translocation capacity in plants such as rice [34] and pak choi [35]. However, other studies reported that exogenous Ca^2+^ supplementation increased Cd content in leaves and stems, as well as translocation coefficients, in rice during both the tillering and maturity stages [36]. This might be attributed to the differences in plant cultivars and environmental conditions. In this study, medium-to-high calcium treatments promoted Cd uptake and translocation (Table 4). This phenomenon is closely related to the regulation of Cd transport-related genes by Ca^2+^ signaling. Cd stress accelerates the synthesis of GSH and PCs in plants. These compounds bind to Cd to form highly soluble complexes, facilitating their transport via the xylem and rapid accumulation in shoots [37]. Concurrently, Ca^2+^ treatment may regulate the physiological activity of Cd transport-related transporters in maize roots and xylem, thereby enhancing Cd translocation from roots to shoots, as evidenced by the highly significant increase in the translocation coefficient (Table 4). Ye et al. [38] found that Ca^2+^ decreased Cd content in rice roots but facilitated Cd translocation from roots to shoots, supporting our results.

This study did not elucidate the specific regulatory mechanisms by which exogenous Ca^2+^ supplementation influenced plant Cd uptake. Further studies should focus on the subcellular distribution of Cd and the responses of related transporter protein genes.

Taken together, our data revealed that Cd stress induced an increase in MDA content in maize seedlings, indicating aggravated oxidative damage that inhibited seedling growth. This study indicated that Ca^2+^ supplementation alleviated the negative impacts of Cd on the biochemistry and growth of maize, and also promoted the absorption of Cd. Therefore, although growth returned to normal, the presence of Cd made the plant unsuitable for human or animal consumption. Exogenous Ca^2+^ supplementation alleviated the inhibitory effects of Cd stress on RL, dry weight, and photosynthetic pigment content, while enhancing the activities of SOD and CAT in leaves. Medium-to-high Ca^2+^ concentrations elevated GSH and AsA contents in seedlings under Cd stress, thereby reducing Cd-induced lipid peroxidation in cell membranes. PCA revealed that the 5 mM Ca^2+^ treatment achieved the highest comprehensive score, thereby optimizing all measured maize parameters. This study not only advances the understanding of the role of Ca^2+^ in plant stress response mechanisms but also provides novel strategies for safe maize production in Cd-contaminated soils, demonstrating significant theoretical innovation and practical value.
